# RACIAL DIFFERENCES IN CHARACTERISTICS AND PERCEPTIONS OF CAREGIVERS TO BLACK AND WHITE OLDER ADULTS

**DOI:** 10.1093/geroni/igz038.747

**Published:** 2019-11-08

**Authors:** Chanee D Fabius, Chanee Fabius, Jennifer Wolff, Judith Kasper

**Affiliations:** Johns Hopkins University, Baltimore, Maryland, United States

## Abstract

Persistent racial differences in health, socioeconomic characteristics, and service utilization of older adults likely result in differential effects for the circumstances and experiences of family and unpaid caregivers. Utilization of community-based services has been found to alleviate caregiver burden, but the extent to which supports and informal help affect race differences in caregiver perceptions (e.g. positive/negative feelings associated with caregiving) and engagement in activities such as religious services, volunteering, or visiting family and friends, is less understood. Using data from the 2015 National Health and Aging Trends Study (NHATS; Round 5) and the National Study of Caregiving (NSOC; Round 2), nationally representative studies of Medicare beneficiaries aged 65 and older and their caregivers, this presentation will discuss the association between sociodemographic characteristics, use of assistance from others or supportive services, and perceived gains, difficulties, and social engagement among caregivers to older black and white adults.

